# Angioleiomyoma of the External Auditory Canal in a Pediatric Patient: A Case Report

**DOI:** 10.1155/crot/1538233

**Published:** 2025-06-15

**Authors:** Wm. Zachary Salter, Kolos K. Nagy, Drew H. Smith, Arif Dauod, Tam Q. Nguyen

**Affiliations:** ^1^School of Medicine, Texas Tech University Health Sciences Center, Lubbock, Texas, USA; ^2^Department of Otolaryngology-Head and Neck Surgery, Texas Tech University Health Sciences Center, Lubbock, Texas, USA; ^3^Department of Pathology, Texas Tech University Health Sciences Center, Lubbock, Texas, USA

## Abstract

Angioleiomyoma (ALM) is a subtype of leiomyoma characterized by vascular involvement alongside the proliferation of smooth muscle cells. These tumors are generally found in the lower limb and rarely occur in the head and neck region. Herein, we present a rare case of ALM of the external auditory canal (EAC) in a 12-year-old female. To our knowledge, this is the sixth case and the second youngest patient reported with ALM occurring in the EAC. Initially, this patient was diagnosed with otitis externa with polypoid change and prescribed a course of Ciprodex. Follow-up CT demonstrated a soft tissue density in the right EAC, consistent with medial canal fibrosis, and an exam under anesthesia with local excision of the mass was scheduled. Surgical findings showed a cartilaginous, firm mass originating from the fissure of Santorini that was carefully excised completely along with an EAC cholesteatoma seen lateral to the tympanic membrane. The foramen of Huschke was uninvolved and the tympanic membrane was intact without perforation. Final pathology confirmed benign ALM. The EAC was packed with floxin-soaked gelfoam and left packed for 2 weeks with instructions for daily floxin drop placement. Once the gelfoam was removed in the clinic, the EAC was found to be healing well and the patient noted improved subjective hearing. There has not been any subsequent recurrence over a period of 5 months. This case documents a rare presentation of an ALM in an extremely rare anatomical position which was managed successfully.

## 1. Introduction

Angioleiomyoma (ALM) is a benign tumor composed of mature smooth muscle cells. ALM most commonly arises in the digestive tract, uterus, or lower limb, with highest incidence in females in their third to sixth decades of life. Leiomyomas of the head and neck can arise if a smooth muscle component is present, including blood vessels (ALM) or arrector pili units (piloleiomyoma) [1].

ALMs of the head and neck are rare, and very few have been reported arising from the external auditory canal (EAC), most of them being in male patients of older age [2]. There has been one pediatric case of ALM in the EAC reported to our knowledge [1]. The etiology of ALMs is largely unknown, but venous stasis, trauma, hamartomatous lesions, or estrogenic hormonal changes have been described as risk factors for its occurrence [3].

## 2. Case Presentation

A 12-year-old female presented to her primary care physician with right purulent otorrhea, pain, and muffled hearing. She had no significant past medical history. The patient denied recent fever and completed a course of Ciprodex and Augmentin. On initial otoscopic examination, there was significant edema of the right ear canal which prevented view of the tympanic membrane, along with scant purulent drainage, ear canal excoriation, and polypoid change.

**Figure 1:**
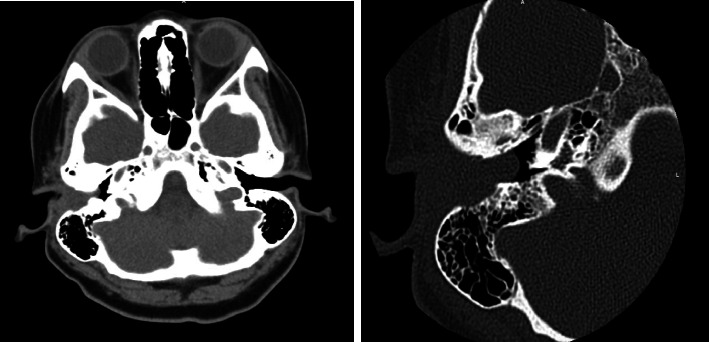
CT demonstrating obstruction of the right EAC by a soft tissue mass without erosion.

Culture of the ear canal revealed pan-sensitive Pseudomonas aeruginosa. Otitis externa with polypoid change was suspected, and the patient was prescribed a course of Ciprodex. Computed Tomography (CT) of the temporal bone demonstrated a soft tissue density in the right EAC, without any obvious erosive changes or signs of remodeling, consistent with medial canal fibrosis (Figure 1). The patient was discharged and scheduled for a follow up.

In the follow up, the patient stated that her ear pain improved and denied pulsatile tinnitus. Otoscopic examination demonstrated purulent drainage, with possible ear canal stenosis and a fibrous, highly vascular mass causing 100% obstruction. Due to its fibrous nature, the mass can be differentiated from a polyp, which primarily exhibits vascular characteristics. Keratin debris was observed behind the mass abutting the TM. The debris was removed and the TM and most of the bony EAC was uninvolved, indicating that the debris was trapped desquamation behind the mass; therefore, further imaging was not warranted. A hearing test demonstrated a type B tympanic membrane with reduced compliance, word recognition 100%, speech reception threshold of 40 dB HL, and mild to moderately severe conductive hearing loss. Because the patient was a child, glomus tumor was less concerning, but an excisional biopsy was planned to rule out soft tissue sarcoma or invasive malignancy. The patient was scheduled for right EAC polyp removal with possible tympanoplasty and canaloplasty.

**Figure 2:**
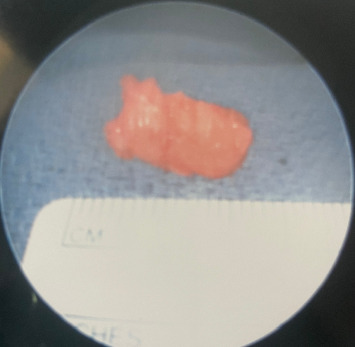
Gross image of postop excised mass.

Intraoperative findings showed a cartilaginous and firm vascular mass with 100% obstruction of the right EAC originating from the fissure of Santorini. The lesion was carefully removed, along with an EAC cholesteatoma lateral to the TM (Figure 2). Medial EAC canal narrowing from the circumferential osteoma was visualized. The Foramen of Huschke was uninvolved, and the TM was intact and without perforation. The EAC was irrigated and suctioned and packed with floxin-soaked gelfoam. The patient was instructed to leave the packing for 2 weeks to administer daily floxin drop placement.

**Figure 3:**
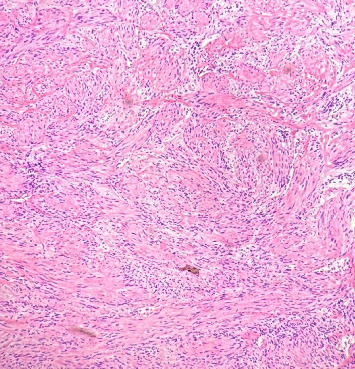
Bland spindle cells in bundles/fascicles with intervening small blood vessels.

**Figure 4:**
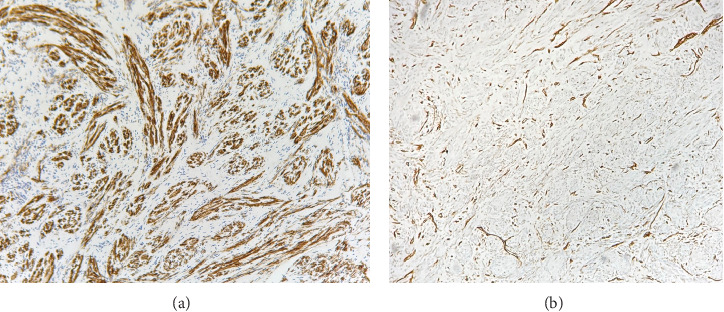
Smooth muscle stain (SMA), highlighting bundles of smooth muscle (a). CD31 (endothelial marker), highlighting intervening vasculature (b).

The resected mass was biopsied perioperatively. Histological analysis demonstrated a neoplasm consisting of whorled spindle cells around thin-walled blood vessels (Figure 3). Immunohistochemical (IHC) staining of the spindle cells was positive for smooth muscle stain (SMA), DOG1, caldesmon, and SMMHC, with a low Ki-67 index (< 1%), and negative for calretinin, desmin, HMB-45, factor XIIIa, pancytokeratin, S100, SOX10, synaptophysin, and CD117 (Figure 4). The lesion's gross morphology, histological appearance, and IHC staining pattern were consistent with ALM of the EAC.

Two weeks later, gelfoam was removed in the clinic, and the EAC was healing well. Patient noted improved subjective hearing. No subsequent recurrence has been noted over a follow up period of 5 months.

## 3. Discussion

This case expands the known manifestations of ALM and adds to the growing number of cases describing its occurrence in the EAC. To our knowledge, only six of these cases have been previously reported, of which only one was in a pediatric patient.

Malignant tumors of the EAC are more common than their benign counterparts, and they most commonly arise as sarcomas, carcinomas, or melanomas [4]. Benign tumors of the EAC most commonly develop from ceruminous glands as adenomas. Schwannomas, hemangiomas, or neuromas are other primitive tumors of the EAC, but these rarely occur. While the differential for soft tissue tumors in the EAC is broad, few are vascularized. Vascularized masses in the EAC warrant a high index of suspicion; therefore, resection and biopsy are necessary to exclude malignancy. ALM is exceedingly rare in general, and only a handful of cases have reported its occurrence in the EAC. In the case a lesion is found in the EAC, malignancy must be excluded due to its atypical and vascular nature, as it poses a risk of extension, bone destruction, and intracranial involvement. In a child, the primary concern for malignancy in the EAC remains soft tissue sarcoma [2, 4].

ALM of the EAC may present with hearing loss, auricular fullness, otalgia, and otorrhea. Thus, it is difficult to diagnose and treat without resection and biopsy [4]. In rare cases, ALM can be asymptomatic and be incidentally noticed during routine physical examination [1].

CT of ALM can show a well-defined soft tissue mass with opacification without erosion of bone or involvement of the middle and inner ear. Otologic examination can demonstrate an opaque, mildly vascularized soft tissue mass, but its visualization may be difficult in some cases [2]. If negative, otomicroscopic investigation may be necessary to visualize the morphologic features ALM can exhibit.

ALMs can be mistaken for medial canal fibrosis, polyp, or cholesteatoma on physical exam, and a high index of suspicion and broad differential is necessary to diagnose this tumor [4]. ALMs are difficult to elucidate from other pathologies of the EAC and are thus rarely diagnosed before surgery without extensive histological examination [5]. Of the reported cases, the majority arises at the posterior wall, but they may occur in other regions such as the anterior and superior walls of the EAC [6].

ALMs typically arise from the tunica media layer of vessels due to its dense smooth muscle component. ALM can be histologically visualized as a region of well-circumscribed and well-differentiated smooth muscle cells arranged in a closely packed manner with intervening vascular spaces that extend in finger-like projections from vascular channels [2, 6]. Hyalinization, calcification, and myxomatous changes can also be seen in some cases [2]. IHC staining can be positive for SMA, desmin, myosin, trichrome, HHF35, calponin, and h-caldesmon, though there may be some variation [2].

Three subtypes of ALM were described by Marimoto in 1973: solid, venous, and cavernous phases. These subtypes can be further grouped into extremity and head tumors, the latter of which are venous in nature, typically painless, and encompass those found in the EAC [5, 7]. The solid type is characterized by close, compacted smooth muscle cell bundles with numerous but small vascular channels. The venous type has thick muscular vascular channels with less compacted and dispersed smooth muscle cell bundles. The cavernous type contains areas of dilated vascular channels with small amounts of smooth muscle cells [4].

Resections remain the primary method of treatment; postauricular, endaural, retroauricular, and mastoid approaches have been described [3]. Following resection, meticulous IHC analysis is necessary to exclude leiomyosarcoma and malignant transformation, both of which, if left undetected, may progress and require emergent treatment [6]. AMLs of EAC generally have good prognosis and have low likelihood of recurrence, even without pharmacologic treatment or radiation therapy [2, 8].

Our findings expand the known predilections of ALM. The occurrence of ALM in the EAC of a pediatric patient has not been reported in great breadth and is exceedingly rare. This case demonstrates the need for a high index of suspicion in lesions involving the EAC and expands the conventional differential diagnosis of lesions involving the EAC in pediatric patients.

## 4. Conclusion

ALM tumors are rarely found in the EAC, and only one previous case has been reported in a pediatric patient. Alongside surgical resection, IHC analysis is necessary to identify ALM and exclude possible malignancy in these peculiar tumors. The increasing number of reported ALMs of the EAC necessitates that physicians maintain a high index of suspicion when dealing with soft tissue masses arising in the EAC. Resection generally resolves symptoms, the prognosis of these tumors is good, and recurrence is rare, but longitudinal care and surveillance are necessary to monitor the possibility of suboptimal outcomes considering the limited amount of literature on this subject.

## Data Availability

Data sharing is not applicable to this article as no new data were created or analyzed in this study.
